# Antibacterial Activities of Methanol and Aqueous Extracts of *Salvadora persica* against *Streptococcus mutans* Biofilms: An In Vitro Study

**DOI:** 10.3390/dj9120143

**Published:** 2021-12-01

**Authors:** Abdulrahman A. Balhaddad, Lamia Mokeem, Mary Anne S. Melo, Richard L. Gregory

**Affiliations:** 1 Department of Restorative Dental Sciences, College of Dentistry, Imam Abdulrahman Bin Faisal University, Dammam 31441, Saudi Arabia; 2 PhD Program in Biomedical Sciences, University of Maryland School of Dentistry, Baltimore, MD 21201, USA; lsmokeem@umaryland.edu (L.M.); mmelo@umaryland.edu (M.A.S.M.); 3 Operative Dentistry Division, Department of General Dentistry, University of Maryland School of Dentistry, Baltimore, MD 21201, USA; 4 Department of Biomedical Science and Comprehensive Care, Indiana University School of Dentistry, Indianapolis, IN 46202, USA; rgregory@iu.edu

**Keywords:** dental caries, oral biofilm, *Salvadora persica*, *Streptococcus mutans*

## Abstract

The use of herbal products in oral hygiene care has a long history, and their use is popular today. A tree stick, named *Salvadora persica* (*S. persica*), is commonly used to remove dental plaque and clean teeth in many countries. In addition, extracts of *S. persica* can be used as a mouthwash, as they demonstrate antimicrobial properties. This study aimed to investigate the antibacterial effect of *S. persica* methanol and aqueous extracts against *Streptococcus mutans* (*S. mutans*) biofilm. A *S. mutans* biofilm formation assay was conducted using different concentrations of *S. persica* methanol or water extracts in tryptic soy broth (TSB) supplemented with 1% sucrose. The biofilm was stained with crystal violet dye, and the absorbance was assessed to examine biofilm formation. One-way analysis of variance (ANOVA) and Tukey tests were used to analyze the results. The *S. persica* methanol extract displayed a significant inhibition (*p* ≤ 0.001) against the *S. mutans* biofilm. The 10 mg/mL concentration of the *S. persica* methanol extract was determined as the minimum biofilm inhibitory concentration (MBIC). The used methanol concentration, mixed with TSB supplemented with 1% sucrose and without the *S. persica* extract, did not inhibit the *S. mutans* biofilm. The *S. persica* aqueous extract did not demonstrate any biofilm inhibition at any concentration (*p* ≥ 0.05). The findings of this study suggest the potential of using *S. persica* methanol extract as a mouthwash or adjunctive to oral hygiene tools.

## 1. Introduction

Dental caries is one of the most prevalent oral diseases around the world. It appears when demineralization factors overcome the remineralization capabilities of the saliva reservoir, resulting in mineral imbalances within the tooth surface [1]. Two microorganisms are among the key pathogens causing dental caries: *Streptococcus mutans* (*S. mutans*) and *Lactobacillus* species [2]. These species can attach to the salivary dental pellicle formed over the tooth surface, utilizing the carbohydrate to produce lactic acid and demineralizing the tooth surface [3]. Various approaches have been investigated as anticaries strategies, aiming to decrease cariogenic biofilm formation via the use of antimicrobial agents [4].

Throughout the history of humankind, herbal remedies have been used to treat many infectious diseases. The validity of these remedies was based on experience and practice, rather than experimental studies or science. Some of these techniques are still utilized as a part of tradition, culture, or religious practices. Miswak is an Arabic word that refers to ‘a chewing stick’ taken from specific plants, mainly the *Salvadora persica* (*S. persica*) plant [5]. Miswak is widely used in Arab countries, India, and Pakistan as a teeth cleaner [6]. Due to the recent interest in non-antibiotic antibacterial approaches, as a result of the increased rate of antibiotic bacterial resistance, several herbal products have been explored for their antibacterial properties. Many of these approaches have targeted cariogenic bacteria [7] and periodontal pathogens [8]. *S. persica* extracts may have antimicrobial potential against several oral fungal and bacterial species, due to multiple antimicrobial compounds within the stick [9].

The popular practice of chewing *S. persica* sticks presents some drawbacks, including the difficulty in accessing the lingual and proximal surfaces of the teeth [10] and the increased risk of causing gingival recession [11]. Alternatively, providing this material as a mouthwash may eliminate such disadvantages. Therefore, several oral products that contain miswak extracts have been introduced on the market, such as Listerine^®^ Miswak mouthwash (Johnson & Johnson Middle East FZ-LLC, Dubai, United Arab Emirates). This mouthwash, in particular, is marketed as an alcohol-free alternative. However, studies have reported conflicting outcomes related to the type of miswak extracts and their relationship to any antibacterial effect [12,13,14]. Considering this point, it is critical to investigate the antibacterial activity of *S. persica* using different concentrations and different extracts.

Therefore, this study aimed to explore the *S. mutans* biofilm inhibition by *S. persica* using two different extracts, aqueous and methanol, in various concentrations. We hypothesized that the *S. persica* concentration and type of extract would affect the antibacterial activities of *S. persica*.

## 2. Materials and Methods

### 2.1. Preparation of the Aqueous and Methanol Extracts

*S. persica* miswak stick was bought from Pakistan (Al-Falah IMPEX, Karachi, Sindh, Pakistan). The *S. persica* stick was ground into powder, and three grams of the miswak powder was mixed with 15 mL of either water or methanol. The extracts were transferred into clean vials and stored at 4 °C for one week, to allow the water and methanol to extract the chemical components of the powder. Then, both extracts were centrifuged at 3000 rpm for 15 min [15]. The supernatant was filtered and diluted using tryptic soy broth (TSB) supplemented with 1% sucrose to obtain different concentrations (0.31, 0.62, 1.25, 2.5, 5, 10, 20, and 40 mg/mL) of the soaked *S. persica* powder. Both extracts were kept at 4 °C, until further use.

### 2.2. Effect of S. persica Extracts on S. mutans Growth

**Figure 1:**
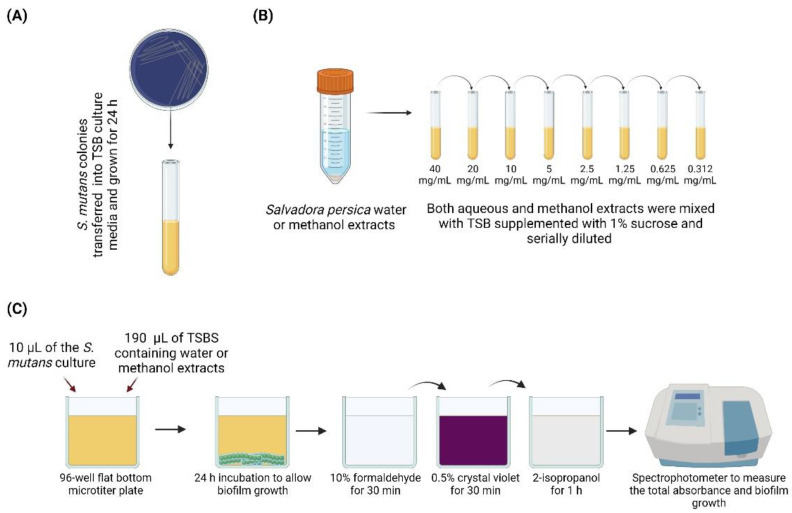
Schematic drawing representing the design of the study. (**A**) *S. mutans* colonies grown in a selective agar plate were transferred into 5 mL of tryptic soy broth (TSB) and grown for 24 h. (**B**) *S. persica* methanol and water extracts were serially diluted to generate several concentrations, ranging from 0.312 to 40 mg/mL. (**C**) 10 µL of the overnight culture was mixed with 190 µL of TSB supplemented with 1% sucrose containing each extract concentration and incubated for 24 h. On the following day, the biofilms were read at 490 nm to measure the biofilm absorbance.

A single *S. mutans* (UA159) colony was isolated from mitis salivarius agar supplemented with bacitracin and placed in 5 mL of TSB (tryptic soy broth) for 24 h (Figure 1A). TSB supplemented with 1% sucrose was mixed with 40 mg/mL of aqueous and methanol extract and serially diluted to create a range of concentrations of *S. persica* aqueous and methanol extracts (Figure 1B). Then, 10 µL of the overnight culture of *S. mutans* (approximately 10^6^ colony-forming units (CFU)/mL) in TSB was treated with 190 µL of 0.31, 0.62, 1.25, 2.5, 5, 10, 20, or 40 mg/mL of *S. persica* aqueous and methanol extracts mixed with TSB supplemented with 1% of sucrose for 24 h in sterile 96-well flat-bottom microtiter plates (Fisher Scientific, Newark, DE, USA) (Figure 1C). The minimum biofilm inhibitory concentration (MBIC) was defined as the lowest concentration that yielded a significant change in the biofilm’s optical density (_OD_), compared to the control [16]. Total absorbance (biofilm and planktonic growth) was measured in a spectrophotometer (SpectraMax 190; Molecular Devices Inc., Sunnyvale, CA, USA) at 595 nm.

The remaining planktonic cells were discarded from the biofilm microtiter plate wells (leaving an attached biofilm), and 200 µL of 10% formaldehyde was added to each well for 30 min to fix the cells. After 30 min, the formaldehyde was removed, the biofilm cells were washed three times with deionized water, and 200 µL of 0.5% crystal violet dye was added to each well to stain the cells for 30 min. The wells were rinsed three times, and 200 µL of 2-isopropanol was placed into each well for 1 h to lyse the cells and extract the crystal violet (Figure 1C). The wells were read with a spectrophotometer at 490 nm, to measure biofilm formation [16]. This study used two control groups: a negative control with only *S. mutans* overnight culture and TSB supplemented with 1% sucrose growth media, and a sterility control group with only TSB growth media, to ensure the absence of any microbial contamination.

### 2.3. Sample Size Calculation and Statistical Analysis

Based on prior studies, the within-group standard deviation of the absorbance measurements for biofilm formation was estimated to be 0.15. Thus, this study had an 80% power to detect a difference at 5% significance level, with 4 samples in each of the 3 repeated experiments. One-way ANOVA and Tukey tests were utilized to compare the effects of miswak extracts on biofilm and total growth. A *p*-value < 0.05 was considered statistically significant.

## 3. Results

### 3.1. Effect of Salvadora persica Water Extract on S. mutans Growth

**Figure 2:**
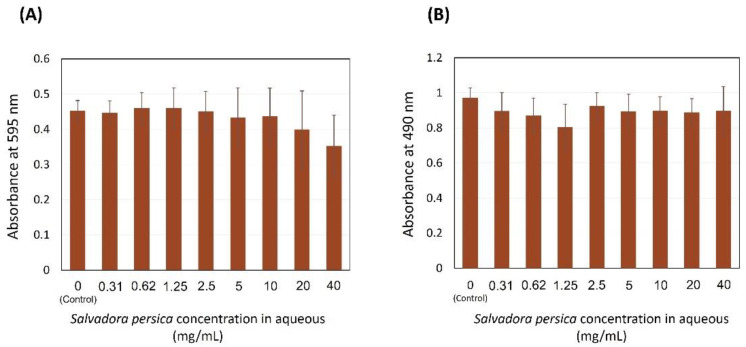
Effect of the *S. persica* aqueous extract on *S. mutans* total absorbance (**A**) and biofilm formation (**B**). Each group consisted of 4 wells, and the experiment was repeated three times (*n* = 12). No differences between groups were detected.

Figure 2A illustrates the different concentrations of *S. persica* extracts assessed against *S. mutans* biofilms. The zero concentration refers to 10 µL of *S. mutans* overnight culture treated with 190 µL of TSB supplemented with 1% sucrose without *S. persica* water extract, which was used as a control in this assay. No inhibition was found in the total growth of the bacteria (*p* = 0.324), using different concentrations of *S. persica* water extract. It can be observed that, with 20 and 40 mg/mL of *S. persica,* there was a minor, but not a significant reduction compared to the control with no treatment. In Figure 2B, no biofilm inhibition is observed at any extract concentration (*p* = 0.135), indicating that *S. persica* water extract might not be suitable for extracting the antimicrobial agents from miswak stick.

### 3.2. Effect of S. persica Methanol Extract on S. mutans Growth

**Figure 3:**
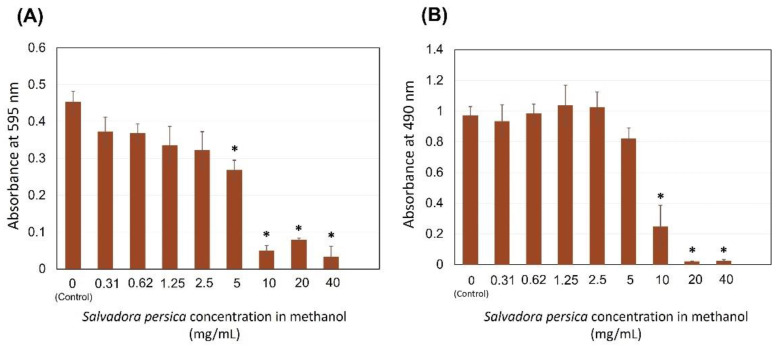
Effect of the *S. persica* methanol extract on *S. mutans* total absorbance (**A**) and biofilm formation (**B**). Each group consisted of 4 wells, and the experiment was repeated three times (*n* = 12). * Asterisks indicate a significant difference compared to the control samples with no treatment.

Figure 3A demonstrates the inhibitory effect of *S. persica* methanol extract on *S. mutans* total growth. The zero concentration refers to 10 µL of *S. mutans* overnight culture treated with 190 µL of TSB supplemented with 1% sucrose without *S. persica* methanol extract, which was used as a control in this assay. A dose-dependent inhibition was observed when the extract concentration was increased. At 5 mg/mL, the *S. persica* started to demonstrate a significant inhibition. More inhibition was observed when the *S. persica* methanol extract concentrations were 10, 20, and 40 mg/mL (*p ≤* 0.001). In Figure 3B, the effect of *S. persica* methanol extract on *S. mutans* biofilm is shown. Concentrations ranging between 0.31 and 5 mg/mL were not associated with major biofilm reduction. However, 10, 20, and 40 mg/mL of *S. persica* were significantly effective in diminishing the biofilm growth (*p ≤* 0.001). The MBIC of the *S. persica* methanolic extract was determined as 10 mg/mL.

**Figure 4:**
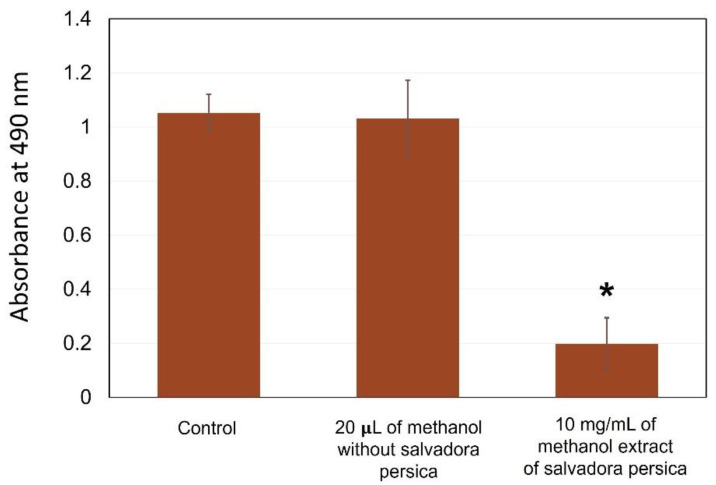
Effect of 20 µL of methanol mixed with 170 µL of tryptic soy broth (TSB) supplemented with 1% sucrose on *S. mutans* biofilm with and without the *Salvadora persica* extract. Using the methanol-TSB supplemented with 1% sucrose mixture did not affect the *S. mutans* biofilm. However, the same methanol concentration containing 10 mg/mL of *S. persica* methanol extract in the TSB supplemented with 1% sucrose significantly inhibited the *S. mutans* biofilm growth. Each group consisted of four wells, and the experiment was repeated three times (*n* = 12). * Asterisk indicates a significant difference compared to the control samples with no treatment.

Methanol is a strong toxic chemical compound. In this study, it was used to extract the chemical components of *S. persica* and then transferred at a low concentration to be tested against *S. mutans*. Therefore, it is possible that the methanol carried out the antibacterial effect observed in Figure 3, rather than the *S. persica* extract. To exclude this possibility, another set of experiments were conducted, where the *S. mutans* biofilm was exposed to 20 µL of methanol mixed with 170 µL TSB supplemented with 1% sucrose. The 20 µL of methanol was prepared with and without *S. persica* (Figure 4). The control refers to 10 µL of *S. mutans* treated with 190 of TSB supplemented with 1% sucrose only. Using methanol alone at a methanol concentration of 10 mg/mL, methanol extract did not harm the *S. mutans* biofilm, as the absorbance value was comparable to the control with no treatment. However, the use of 10 mg/mL of the *S. persica* methanol extract was associated with a significant biofilm inhibition (*p ≤* 0.05). Such results indicate that the biofilm inhibition was dependent on the *S. persica* methanol extract.

## 4. Discussion

Herbal medicine has been the first choice, if not the only, method of health and oral care in large populations, mainly in developing countries. Based on the popular belief that chewing sticks can efficiently clean the teeth, several investigators have directed their research to explore their antibacterial properties [17]. With the limitation of an in vitro study, our results demonstrate that the miswak–methanol extract was able to inhibit the biofilm formation of *S. mutans*. The MBIC was reported as 10 mg/mL. On the contrary, the miswak water extract was not effective in diminishing the growth of *S. mutans*. Our results indicated that the type of extract and its concentration are essential factors to achieve an antimicrobial effectiveness with *S. persica*.

The antimicrobial activities of *S. persica* are attributed to the release of chemical compounds, such as isolated benzyl isothiocyanate (BITC), when the stick is rubbed against the tooth surface [18]. BITC is recognized as a wide-spectrum bactericidal material and can restrict the acid production and the growth of *S. mutans* [5]. In addition, sulfur compounds were found in high quantity in miswak, as antimicrobial materials [5,9,19]. Besides, the essential oil in miswak extracts might increase the buffering capacity of the saliva or increase the antimicrobial activity [5,9,19]. In addition, the presence of other substances such as β-sitosterol, chlorides, salvadourea, organic compounds, piperidine derivatives, glycosides, and flavonoids has been reported [20]. Furthermore, *S. persica* was found to have a considerable amount of fluoride, calcium, and phosphorous, contributing to the remineralization process [5,9,19].

Our findings corroborate the results observed previously in the literature regarding the antimicrobial effects of miswak. In one in vitro study, *S. mutans* biofilm formation was developed on orthodontic brackets and exposed to chlorhexidine and two *S. persica* miswak extracts, hexane, and ethanol extracts [21]. The results exhibited no significant differences regarding the absorbance values of the three groups [21]. Moreover, some epidemiological studies found a lower caries incidence among miswak users than toothbrush users [22,23]. In one investigation, *S. persica* extract was found to be more potent than other chewing stick extracts, from *Azadirachta indica* and *Mangifera indica*, in inhibiting *S. mutans* growth [24]. The capability of *S. persica* to compromise the quorum sensing of *S. mutans* was also demonstrated [12]. Moreover, *S. persica* at 50% concentration was found to be effective for inhibiting other streptococcus species, such as *Streptococcus faecalis* and *Streptococcus sanguis* [25].

Most studies used water or alcoholic solutions to extract miswak components. However, there were conflicting results regarding this, and some studies indicated that methanol extracts were more efficient [12,13], while others reported that water extraction was more potent [14]. One study investigated the effect of five different extracts, methanol, ethanol, chloroform, acetone, and aqueous extracts [12]. It was found that the methanol group reduced the biofilm formation by 87.92%, more than any other extract. However, the other extracts showed a significant inhibition, as the lowest inhibition rate was associated with aqueous extract, at 58.68% [12]. In this study, it seems that methanol was more effective in extracting the bioactive components of *S. persica.* Such observations may suggest trying other alcoholic solutions to dissolve *S. persica* components.

The limited spectrum of *S. persica* was reported in other studies [26,27]. It was found that *S. persica* miswak extract could have an antimicrobial effect against *S. mutans,* but not against *Lactobacillus* [26]. In addition, Almas et al. conducted a study regarding the efficiency of nine non-alcohol mouthrinses against *Streptococcus faecalis* (*S. faecalis*), *Streptococcus pyogenes* (*S. pyogenes*), *S. mutans*, *Candida albicans* (*C. albicans*), *Staphylococcus aureus* (*S. aureus*)*,* and *Staphylococcus epidermidis* (*S. epidermidis*) [27]. The authors concluded that miswak extract has a minimal antimicrobial activity in comparison to other commercial mouthrinses. The reported limited effectiveness of herbal chewing sticks could be due to the lack of well-designed protocols using this material. In the literature, several concentrations and different extract solutions were discussed [12,13,14], which may explain the conflicting outcomes and emphasize the need to optimize and develop a well-designed product/protocol for use in daily oral hygiene practice.

In clinical practice, many oral hygiene products contain miswak extracts as the main bioactive ingredient. Several kinds of miswak toothpaste and mouthwashes are available on the market. Some in vivo investigations demonstrated the ability of commercially available *S. persica* mouthwashes (Persica™) to reduce the load of *S. mutans* [28], *Enterococcus faecalis* (*E. faecalis*), and *C. albicans* [29]. Furthermore, miswak ingredient-containing toothpaste was found to eliminate dental plaque and reduce the risk of gingivitis [30]; even though these data demonstrate the potent activities of miswak-containing products, more randomized clinical trials are needed to optimize and increase their effectiveness [31].

Various commercial mouthwashes contain alcohol in their contents. Mouthwashes containing alcohol and their possibility of causing soft tissue irritation and oral cancer have been extensively discussed in the dental literature [32]. Despite the existing controversy, there is no clear evidence that mouthwashes containing alcohol cause oral cancer [32]. In our study, we used methanol to extract the chemical components of *S. persica*. The concentration was very low, so it did not harm the *S. mutans* biofilm (Figure 4). Therefore, it is unlikely that the used methanol concentration would harm the oral tissues. Future investigations examining the relation between alcohol in mouthwashes and oral cancer are required, to validate or exclude alcohol use in oral hygiene products.

The effect of *S. persica* on biofilm formation in this study could be underestimated for two reasons. First, mechanical removal of dental plaque was not achieved in the study. Dental plaque is the main reservoir for cariogenic pathogens, to initiate the demineralization process. Therefore, rubbing the *S. persica* stick against the tooth structure will release antimicrobial chemical components in the oral cavity and remove the dental plaque mechanically. Many studies stated the ability of miswak sticks to reduce plaque scores in the oral cavity [10,33], while some reported no significant differences between miswak sticks and toothbrushes in reducing plaque scores [34,35]. Second, chewing miswak can increase salivary stimulation, which might increase the buffering capacity inside the oral cavity [19].

Oral health care providers should be careful in interpreting the data of this study. Toothbrushes, pastes, and flosses are the standards of oral care, while the use of other products such as mouthwashes, waterpicks, and proxabrushes are considered complementary tools. Patients should also consider consulting oral health care providers in using other techniques to maintain their oral hygiene status. For instance, the aggressive use of miswak sticks may harm the gingival tissues, causing gingival irritation [11]. Therefore, monitoring oral care techniques is critical for achieving an optimum hygiene practice, without compromising surrounding tissues.

## 5. Conclusions

The results of this study demonstrated the ability of *S. persica* methanol extract at a 10 mg/mL concentration to inhibit *S. mutans* biofilm formation, highlighting its potential for use as a mouthwash. Future in vivo investigations are required to confirm the clinical benefits of using *S. persica* extract as a mouthwash to prevent plaque formation on teeth.

## Data Availability

The data set generated and analyzed in this study is available upon reasonable request from the corresponding author.

## References

[1] Balhaddad A.A., Kansara A.A., Hidan D., Weir M.D., Xu H.H.K., Melo M.A.S. (2019). Toward Dental Caries: Exploring Nanoparticle-Based Platforms and Calcium Phosphate Compounds for Dental Restorative Materials. Bioact. Mater..

[2] Peterson S.N., Snesrud E., Liu J., Ong A.C., Kilian M., Schork N.J., Bretz W. (2013). The Dental Plaque Microbiome in Health and Disease. PLoS ONE.

[3] Bowen W.H., Koo H. (2011). Biology of *Streptococcus mutans*-Derived Glucosyltransferases: Role in Extracellular Matrix Formation of Cariogenic Biofilms. Caries Res..

[4] Skeie M.S., Klock K.S. (2018). Dental Caries Prevention Strategies among Children and Adolescents with Immigrant- or Low Socioeconomic Backgrounds- Do They Work? A Systematic Review. BMC Oral Health.

[5] Halawany H.S. (2012). A Review on Miswak (*Salvadora persica*) and Its Effect on Various Aspects of Oral Health. Saudi Dent. J..

[6] Dahiya P., Kamal R., Luthra R.P., Mishra R., Saini G. (2012). Miswak: A Periodontist’s Perspective. J. Ayurveda Integr. Med..

[7] Wassel M.O., Khattab M.A. (2017). Antibacterial Activity against *Streptococcus mutans* and Inhibition of Bacterial Induced Enamel Demineralization of Propolis, Miswak, and Chitosan Nanoparticles Based Dental Varnishes. J. Adv. Res..

[8] Sofrata A.H., Claesson R.L.K., Lingström P.K., Gustafsson A.K. (2008). Strong Antibacterial Effect of Miswak against Oral Microorganisms Associated with Periodontitis and Caries. J. Periodontol..

[9] Haque M.M., Alsareii S.A. (2015). A Review of the Therapeutic Effects of Using Miswak (*Salvadora persica*) on Oral Health. Saudi Med. J..

[10] Gazi M., Saini T., Ashri N., Lambourne A. (1990). Meswak Chewing Stick versus Conventional Toothbrush as an Oral Hygiene Aid. Clin. Prev. Dent..

[11] Eid M.A., Selim H.A., al-Shammery A.R. (1991). The Relationship between Chewing Sticks (Miswak) and Periodontal Health. 3. Relationship to Gingival Recession. Quintessence Int..

[12] Al-Sohaibani S., Murugan K. (2012). Anti-Biofilm Activity of *Salvadora persica* on Cariogenic Isolates of *Streptococcus mutans*: In Vitro and Molecular Docking Studies. Biofouling.

[13] Siddeeqh S., Parida A., Jose M., Pai V. (2016). Estimation of Antimicrobial Properties of Aqueous and Alcoholic Extracts of *Salvadora persica* (Miswak) on Oral Microbial Pathogens—An Invitro Study. J. Clin. Diagn. Res..

[14] Al-Bayati F.A., Sulaiman K.D. (2008). In Vitro Antimicrobial Activity of *Salvadora persica* L. Extracts Against Some Isolated Oral Pathogens in Iraq. Turk. J. Biol..

[15] Darmani H., Nusayr T., Al-Hiyasat A.S. (2006). Effects of Extracts of Miswak and Derum on Proliferation of Balb/C 3T3 Fibroblasts and Viability of Cariogenic Bacteria. Int. J. Dent. Hyg..

[16] Balhaddad A.A., Melo M.A.S., Gregory R.L. (2019). Inhibition of Nicotine-Induced *Streptococcus mutans* Biofilm Formation by Salts Solutions Intended for Mouthrinses. Restor. Dent. Endod..

[17] Wu C.D., Darout I.A., Skaug N. (2001). Chewing Sticks: Timeless Natural Toothbrushes for Oral Cleansing. J. Periodontal Res..

[18] Sofrata A., Santangelo E.M., Azeem M., Borg-Karlson A.-K., Gustafsson A., Pütsep K. (2011). Benzyl Isothiocyanate, a Major Component from the Roots of *Salvadora persica* Is Highly Active against Gram-Negative Bacteria. PLoS ONE.

[19] Hattab F.N. (1997). Meswak: The Natural Toothbrush. J. Clin. Dent..

[20] Galletti G.C., Chiavari G., Kahie Y.D. (1993). Pyrolysis/Gas Chromatography/Ion-Trap Mass Spectrometry of the ‘Tooth Brush’ Tree (*Salvadora persica* L.). Rapid Commun. Mass Spectrom..

[21] Halawany H.S., Abraham N.B., Siddiqui Y.M., Balto H.A., Jacob V. (2016). Antimicrobial Efficacy of *Salvadora persica* Extracts on a Monospecies Biofilm on Orthodontic Brackets In Vitro. Oral Health Prev. Dent..

[22] Younes S.A., El-Angbawi M.F. (1982). Dental Caries Prevalence in Intermediate Saudi Schoolchildren in Riyad. Community Dent. Oral Epidemiol..

[23] Sathananthan K., Vos T., Bango G. (1996). Dental Caries, Fluoride Levels and Oral Hygiene Practices of School Children in Matebeleland South, Zimbabwe. Community Dent. Oral Epidemiol..

[24] Elangovan A., Muranga J., Joseph E. (2012). Comparative Evaluation of the Antimicrobial Efficacy of Four Chewing Sticks Commonly Used in South India: An in Vitro Study. Indian J. Dent. Res..

[25] Almas K., Al-Bagieh N.H., Akpata E.S. (1997). In Vitro Antimicrobial Effects of Extracts of Freshly Cut and 1-Month-Old Miswak (Chewing Stick). Biomed. Lett..

[26] Almas K., Al-Zeid Z. (2004). The Immediate Antimicrobial Effect of a Toothbrush and Miswak on Cariogenic Bacteria: A Clinical Study. J. Contemp. Dent. Pract..

[27] Almas K., Skaug N., Ahmad I. (2005). An in Vitro Antimicrobial Comparison of Miswak Extract with Commercially Available Non-Alcohol Mouthrinses. Int. J. Dent. Hyg..

[28] Salehi P., Sh M.D. (2006). Comparison of the Antibacterial Effects of Persica Mouthwash with Chlorhexidine on *Streptococcus mutans* in Orthodontic Patients. DARU J. Pharm. Sci..

[29] Bafti L.S., Rad M., Soormaghi M.S., Rezaei M. (2013). An in Vivo Evaluation of Antimicrobial Effects of Persica Herbal Mouthwash on Candida Albicans and Enterococcus Faecalis. J. Oral Health Oral Epidemiol..

[30] Azaripour A., Mahmoodi B., Habibi E., Willershausen I., Schmidtmann I., Willershausen B. (2017). Effectiveness of a Miswak Extract-Containing Toothpaste on Gingival Inflammation: A Randomized Clinical Trial. Int. J. Dent. Hyg..

[31] Martin K.W., Ernst E. (2003). Herbal Medicines for Treatment of Bacterial Infections: A Review of Controlled Clinical Trials. J. Antimicrob. Chemother..

[32] Werner C.D.A., Seymour R.A. (2009). Are Alcohol Containing Mouthwashes Safe?. Br. Dent. J..

[33] Norton M.R., Addy M. (1989). Chewing Sticks versus Toothbrushes in West Africa. A Pilot Study. Clin. Prev. Dent..

[34] Al-Otaibi M., Al-Harthy M., Gustafsson A., Johansson A., Claesson R., Angmar-Månsson B. (2004). Subgingival Plaque Microbiota in Saudi Arabians after Use of Miswak Chewing Stick and Toothbrush. J. Clin. Periodontol..

[35] Eid M.A., al-Shammery A.R., Selim H.A. (1990). The Relationship between Chewing Sticks (Miswak) and Periodontal Health. 2. Relationship to Plaque, Gingivitis, Pocket Depth, and Attachment Loss. Quintessence Int..

